# Effects of Three Pesticides on the Earthworm *Lumbricus terrestris* Gut Microbiota

**DOI:** 10.3389/fmicb.2022.853535

**Published:** 2022-03-29

**Authors:** Angelika Astaykina, Rostislav Streletskii, Mikhail Maslov, George Krasnov, Victor Gorbatov

**Affiliations:** ^1^Soil Science Faculty, Lomonosov Moscow State University, Moscow, Russia; ^2^Engelhardt Institute of Molecular Biology, Russian Academy of Sciences, Moscow, Russia; ^3^Centre for Ecopesticides Research, LLC, Moscow, Russia

**Keywords:** pesticides, next-generation sequencing, earthworm, gut microbiota, bacterial biodiversity

## Abstract

Earthworms play a vital role in the terrestrial ecosystem functioning and maintenance of soil fertility. However, many pesticides, for example, imidacloprid, benomyl, and metribuzin that are world-widely used in agriculture, may be potentially dangerous to earthworms. At the same time, standard tests for pesticides acute and chronic toxicity do not reflect all aspects of their negative impact and might not be enough sensitive for effective assessment. In this paper, we studied the effects of non-lethal concentrations of imidacloprid, benomyl, and metribuzin on the gut bacterial community of *Lumbricus terrestris* using high-throughput sequencing approach. We found that pesticides reduced the total bacterial diversity in the earthworm’s gut even at the recommended application rate. Under the applied pesticides, the structure of the gut prokaryotic community underwent changes in the relative abundance of the phyla Proteobacteria, Actinobacteria, Acidobacteria, Planctomyces, Verrucomicrobia, and Cyanobacteria, as well as the genera *Haliangium, Gaiella, Paenisporosarcina, Oryzihumus, Candidatus Udaeobacter*, and *Aquisphaera*. Moreover, the pesticides affected the abundance of *Verminephrobacter*—the earthworms’ nephridia specific symbionts. In general, the negative impact of pesticides on bacterial biodiversity was significant even under pesticides content, which was much lower than their acute and chronic toxicity values for the earthworms. These results highlighted the fact that the earthworm’s gut microbial community is highly sensitive to soil contamination with pesticides. Therefore, such examination should be considered in the pesticide risk assessment protocols.

## Introduction

According to the Food and Agriculture Organization (FAO), more than 4 million tons of pesticides are used in the world annually;^1^ when released into the environment, most of them might pollute air, soil, ground and surface waters, as well as pose a threat to non-target organisms (Van der Werf, 1996; Carriger et al., 2006; Duke, 2017). Pesticides, even at concentrations not exceeding the Maximum Residue Levels (MRLs), lead to a decrease in biodiversity (Hole et al., 2005; Isenring, 2010; Beketov et al., 2013), in particular, soil microbial biodiversity (Lo, 2010; Puglisi, 2012; Fenner et al., 2013; Astaykina et al., 2020). Moreover, pesticides can change the activity of soil microorganisms. For instance, the fungicide chlorothalonil and insecticide chlorpyrifos have been inhibited dehydrogenase, catalase, urease and acid phosphatase activities (Jastrzȩbska, 2011; Baćmaga et al., 2018).

Earthworms as important representatives of soil invertebrates are often called ‘ecosystem engineers’ because their activity modifies physicochemical and biological properties of the habitat (Jones et al., 1994). Depending on the ecological-trophic group, earthworms can contribute to additional migration of pollutants in the soil (Kuzyakov and Blagodatskaya, 2015). For example, *Lumbricus terrestris* which belongs to the anecic earthworms (Bouché, 1977; Lavelle et al., 1989) can transport pesticides from the surface to the mineral horizons. *L. terrestris*, unlike the well-studied compost worm *Eisenia fetida*, is widespread in the arable horizons of most world soils. Furthermore, *L. terrestris* appeared to be a more sensitive species than *E. fetida* in the pesticide toxicity tests (Pelosi et al., 2013). Moreover, due to the structural features, such as the greater length of the intestinal tract, digestion in *L. terrestris* takes up to 6 h (Nechitaylo et al., 2010), consequently, when pesticides enter the digestive tract of *L. terrestris*, they are able to have long-term effects on their gut bacteria.

There are plenty of studies devoted to the analysis of the taxonomic composition of the earthworm’s gut microbial community (Horn et al., 2003; Wüst et al., 2011; Sapkota et al., 2020), which usually differ from bacteria isolated from soils or composts (Byzov et al., 2009). The diversity and structure of bacteria in the earthworms’ gut can vary depending on the ecological-trophic group of lumbricid (Egert et al., 2004). However, the study of the pesticide impact on the microbial biodiversity in the intestinal tracts of soil invertebrates remains practically out of sight of researchers, although this locus is generally recognized as a hotspot of microbial activity. For example, it was discovered that the insecticide fipronil may inhibit the growth of *Eudrilus eugeniae* gut bacteria plated on a sterile nutrient agar media (Salokhe and Deshapande, 2014). Besides, Kavitha et al. (2019) noted that organophosphate insecticide monocrotophos led to a reduction of the bacterial and fungal biodiversity and abundance in the earthworm *Lampito mauritii* gut. In addition, the herbicide glyphosate affected decrease of the microbial α-diversity in the earthworms *Alma millsoni*, *Eudrilus eugeniae* and *Libyodrilus violaceus* gut, while the relative abundance of *Enterobacter* spp. *DHL-02, Pseudomonas putida, Pantoea agglomerans* and *Pseudomonas taiwanensis* increased (Owagboriaye et al., 2021). Based on the results of molecular genetic analysis using high-throughput sequencing of the 16S RNA gene, it was found that the herbicide fomesafen might reduce bacterial diversity and shift the bacterial community structure of *Pheretima guillelmi* gut (Chang et al., 2021). Nonetheless, there are no studies which examine the effects of pesticides on the gut microbiota of *L. terrestris*, one of the most common earthworm species. Undoubtedly, this kind of research is important not only from the ecotoxicology perspective, but also for isolating bacteria which degrade pesticides (Sun et al., 2020).

Thus, based on the previous studies, we hypothesized that (1) pesticides might impact on the earthworm *L. terrestris* indirectly, through changing the gut microbial community taxonomic structure; (2) pesticides even at the recommended application rate may reduce the earthworm’s gut microbial diversity; (3) the gut microbial community relatively quickly response to the pesticide application; (4) bacteria whose relative abundance increases under the impact of pesticides, can be recommended for further research in order to isolate strains that degrade pesticides. To test these hypotheses, we set up a laboratory incubation experiment to study the relationship between pesticide-contaminated soil and earthworm gut microbiota. The cutting-edge approach of total DNA metabarcoding allowed us to assess the taxonomic diversity of gut-associated bacteria without isolation and cultivation of microorganisms which guarantees the novelty of the obtained results.

## Materials and Methods

### Soil and Earthworms Collection

For the laboratory experiment soil samples from the surface (0–10 cm) horizon of Umbric Albeluvisols (IUSS Working Group WRB, 2015) were taken in the Odintsovo district of the Moscow region (55°41′ N, 38°05′ E). The experimental site has been fallowed and has not been treated with pesticides and fertilizers over the past 5 years. Five separate soil samples were randomly selected (average distance between sampling points was 2 m) using a shovel. Large plant residues, live rhizomes, and roots were directly removed during collection. In the laboratory, the soil samples were dried at room temperature, sieved (*<*1 mm) and homogenized. The main properties of the soil are presented in Supplementary Table S1.

Mature specimens of the earthworm *L. terrestris* (Linnaeus, 1758) were collected on the same soil site. Earthworms were maintained at a constant temperature of +17 ± 1°C in the dark for 2 weeks before the start of the experiment in soil containing fresh litter of *Acer platanoides* as a food source.

### Pesticides

For the experimental treatment we chose three pesticide formulations that were produced by «Avgust» Inc. (Russia): 700 g/kg metribuzin (herbicide), 200 g/L imidacloprid (insecticide), and 500 g/kg benomyl (fungicide). All actives were > 98.0% pure and met international standards Imidacloprid {4,5-dihydro-N-nitro-1-[(6-chloro-3-pyridyl)-methyl]-imidazolidin-2-ylene-amine} is a highly effective worldwide used neonicotinoid insecticide (Tišler et al., 2009), which is very stable in soil (DT_50_ > 120 days; PPDB: Pesticide Properties DataBase, 2016) and may have long-term effects on non-target species. Metribuzin [4-amino-6-tert-butyl-3-methylthio-1,2,4-triazin-5(4H)-one] is a triazinone herbicide, which has been widely used in agriculture for several decades. Benomyl [N-[1-(butylcarbomoyl)-benzoimidazolyl-2]-0-methylcarbamate] is a systemic benzimidazole fungicide used to control fungal diseases in agriculture, forestry and veterinary. Benomyl, as well as its main metabolite carbendazim are pesticides stable for degradation in soil (DT_50_ = 61–120 days; PPDB: Pesticide Properties DataBase, 2016). The main properties of these pesticides are provided in Supplementary Table 2S. Imidacloprid, benomyl and metribuzin can be used together for the comprehensive protection of cereals, sugar beets and potatoes. Application of these pesticides includes both seed dressing and soil surface spraying before sowing the seeds so that virtually all the applied pesticide dose enters the soil and is not intercepted by the crop.

### Experimental Design

The laboratory incubation experiment was carried out in four replicates at a constant temperature (+17 ± 1°C) in glass flasks covered with a perforated film to prevent water loss. The mass of moist soil (60% WHC) in each vessel was 500 g. The soil moisture was controlled by the weight method every 2 days.

**Figure 1:**
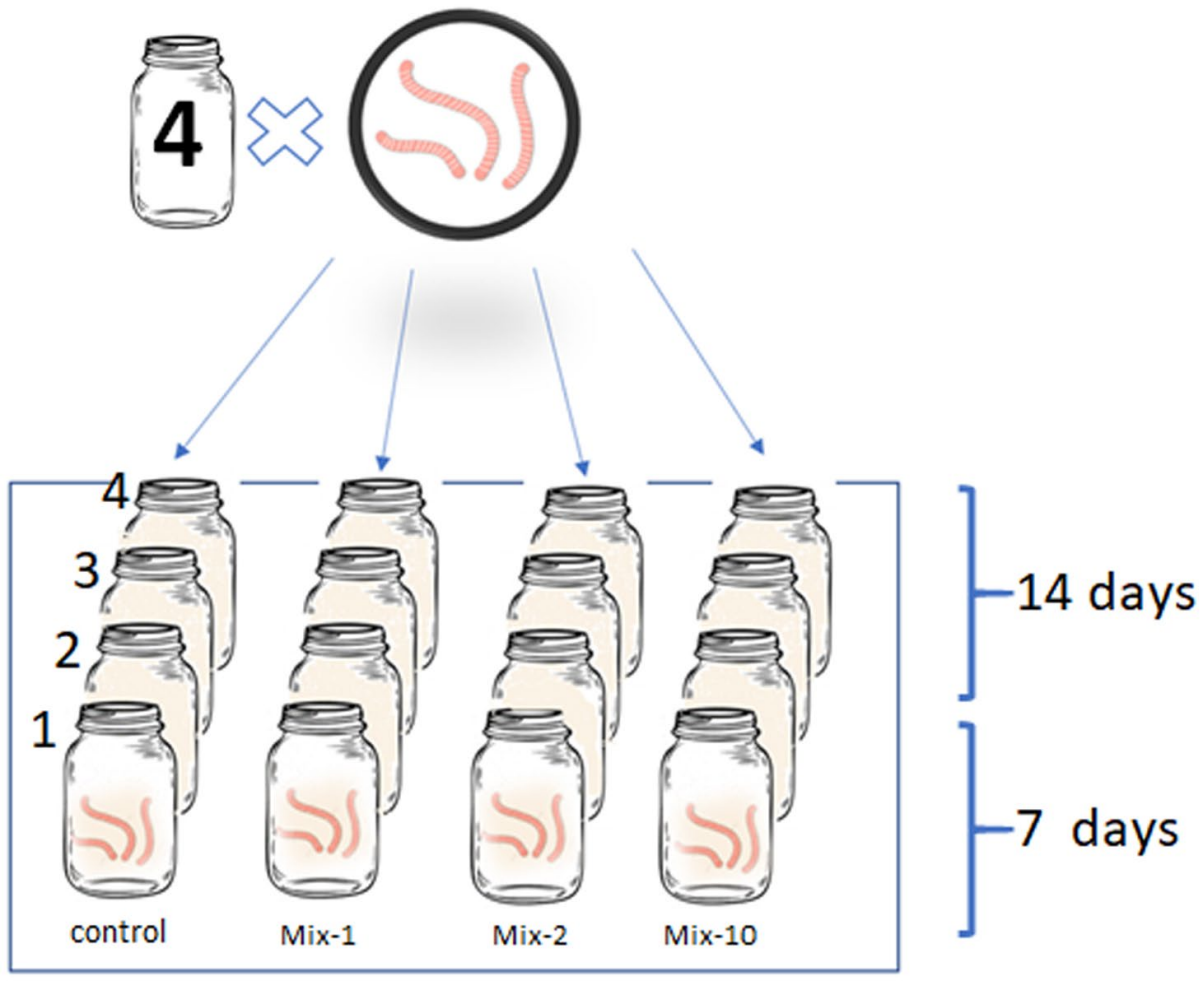
Illustration of the experimental design.

The pesticides were applied according to the manufacturer’s recommended application rates: 0.98 kg/ha metribuzin, 0.02 kg/ha imidacloprid, and 1.5 kg/ha benomyl. We also tested 2-fold and 10-fold application rates that simulated the worst-case scenarios. As was previously mentioned, imidacloprid and benomyl are stable pesticides, therefore, there are likely to accumulate in soil after many years of application on the same site. For a more thorough distribution of pesticides in the soil sample, initially we mixed the pesticide with 20 g of clear sand, and then the already treated sand was thoroughly mixed with the soil aliquot (500 g). In the control, the soil sample was mixed with untreated sand. In total, four variants of the experiment were carried out: a control and a mixture of three pesticides at the manufacturer’s recommended application rates as well as the 2-fold and 10-fold application rates. In each vessel, we placed three specimens of *L. terrestris* (Figure 1). According to the OECD Guidelines for the Testing of Chemicals (OECD, 1984) the duration of the experiment was 14 days. To analyze the intestinal prokaryotic community, the worms were collected after 7 and 14 days, respectively.

### Isolation and Cleaning of Earthworms’ Guts

**Figure 2:**
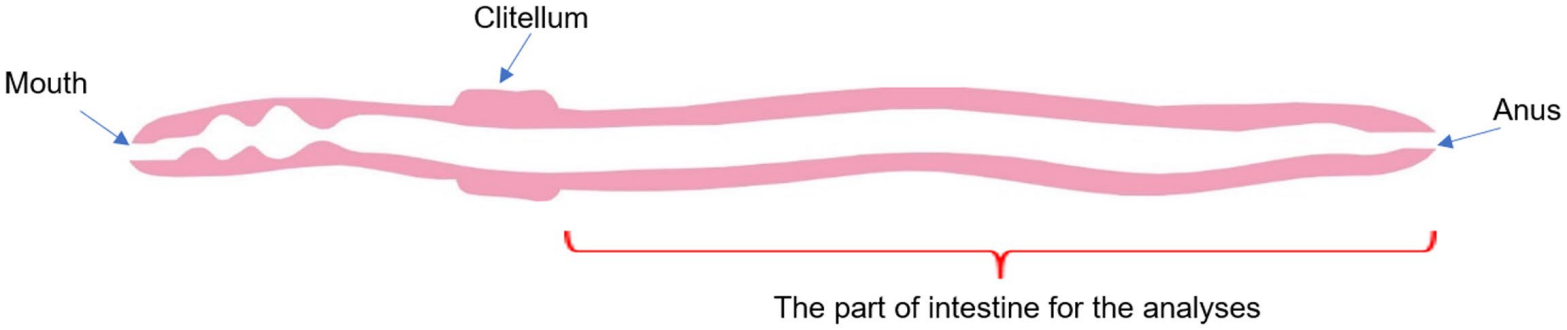
Schematic diagram of the *Lumbricus terrestris* digestive tract.

Before isolating the intestinal tract, the earthworms were washed and kept on wet filter paper for 24 h at a temperature of 6°C–7°C. The earthworms were anesthetized with hot water (100°С) for 3 s and then washed with 70% ethanol. To isolate guts, the worms were frozen on a freezer table (Peltier element) to −16°C and were dissected with a sterile scalpel immediately after defrosting, avoiding repeated freezing–thawing (Byzov et al., 2015). For a molecular genetic analysis, a section of the digestive tract was taken from the clitellum to the anus (Figure 2). The guts cut out in this way were placed in Eppendorf tubes containing 1 ml of sterile Milli-Q quality water and were centrifuged for 10 min at 10,000 *g*. The supernatant was removed and the obtained isolates of microorganisms were stored at −80°C until the subsequent isolation of total DNA.

### Isolation of Total DNA and DNA Metabarcoding

The extraction of total DNA from a 0.5 mg sample of the earthworm’s intestines was performed using the FastDNA SPIN Kit for Soil (MP Biomedicals, United States) according to the manufacturer’s protocol. The isolated DNA extracts were stored at −20°C.

The samples of the isolated DNA were 500-fold diluted. Amplification of the V4 variable region of the 16S rRNA gene was carried out in one round using forward and reverse primers 515F (5′-GTGCCAGCMGCCGCGGTAA-3′) and 806R (5′-GGACTACHVGGGTWTCTAAT-3′) with two-index multiplexing of the samples (Fadrosh et al., 2014). These primers are specific both to bacteria and to archaea. The PCR products were purified using the Cleanup Mini kit (Evrogen, Russia) for DNA isolation from reaction mixtures. The concentration of the obtained 16S rRNA libraries in the solution was measured on a Qubit fluorometer (Invitrogen, United states) using the Quant-iT dsDNA High-Sensitivity Assay Kit. The purified amplicons were mixed equimolarly depending on the concentration. The quality of the library prepared for sequencing was assessed by agarose gel electrophoresis. Further, sample preparation and sequencing of the pooled sample was carried out using the MiSeq Reagent Kit v2 (500 cycles) and an MiSeq sequencer (Illumina, United States).

### Bioinformatics and Statistical Analyses

Bioinformatics analysis was performed as described in the work by Fadrosh et al. (2014). We used the DADA2 package for R, including error correction, inferring ribosomal sequencing variants (RSVs) separately for forward and reverse reads, elimination of chimeric RSVs and, finally, merging forward and reverse RSVs. The obtained average RSV length was about 253 bp (with minimal variability) for bacterial 16S V4 fragments. In contrast to OTUs (operation taxonomic units), the analysis based on RSVs which are also referred to as amplicon sequence variants (ASVs) or exact sequence variants (ESVs) does not imply merging of closely related amplicon variants (<3% differences) into a single sequence (i.e., OTU) and therefore can identify single-nucleotide differences between species (Callahan et al., 2017).

For taxonomic annotation of the obtained RSV sequences, we also used the DADA2 package supplied with the Silva database (version 138) for bacterial communities. RSVs annotated as chloroplasts, mitochondria, Cercozoa, etc. were removed. The resulting read count data were normalized between the samples using the read count annotated at the domain (kingdom) level.

The rest of the analysis was also conducted in the R environment (version 3.6.3). To assess α-diversity, we calculated the Shannon, Chao1 and ACE indices using the fossil 0.4.0 and vegan 2.5–6 packages. When calculating these indices, the read count data were rarefied to match the sample with the minimum number of reads. For the beta diversity analysis, we used Bray-Curtis metric. The rest of the analyses and visualization were performed using the phyloseq 1.30.0, plotly 4.9.2.2, phytools 0.7–47, pheatmap 1.0.12, and ggplot2 3.3.3 packages. To visualize differences between the samples, we applied the metrical and non-metrical multidimensional scaling (MDS). The association analysis between time periods and taxon abundance was performed using the Spearman and Pearson correlations. For non-paired comparison of the two groups, we used the multiple *t*-tests as well as the Mann–Whitney and Pearson tests, and for paired comparisons, we used the Wilcoxon test. The FDR was calculated using the Benjamini–Hochberg adjustment for the obtained value of *p*s. All analyses were carried out in the R environment. For all types of the statistical analysis, the difference was considered significant at a significance level of value of *p* < 0.05.

## Results

### Structure and Composition of Microbial Community of *Lumbricus terrestris* Gut With or Without Pesticide Impact

The study was based on 1,287,366 high-quality reads of 16S rRNA gene amplicons. In total, 208 bacterial genera from 45 classes that belong to 21 prokaryotic phyla were identified in the gut of *L. terrestris*.

**Figure 3:**
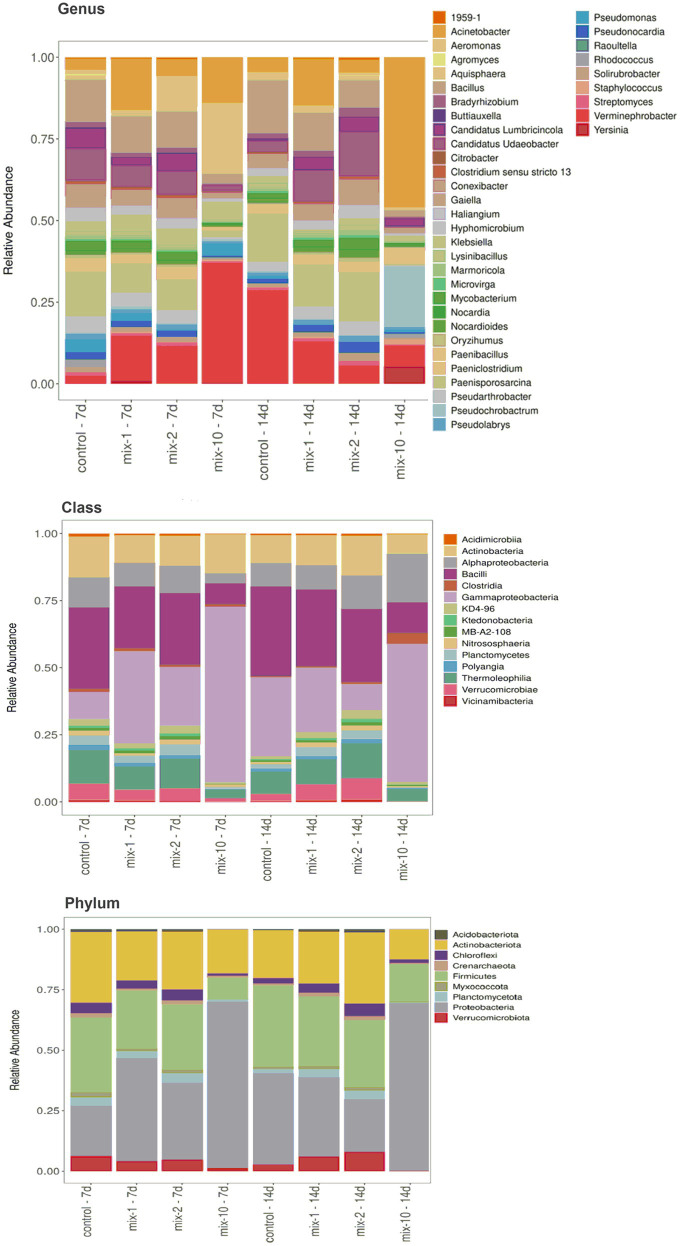
The structure of the prokaryotic community of the intestinal tract of *L. terrestris* at the levels of phyla, classes and genera (*N* = 2). Legend: mix indicates pesticides in a mixture; 1/2/10 is the recommended, 2-fold and 10-fold pesticide application rates; 7 day/14 day is the incubation time. The relative abundance is shown for taxa with read counts >0.3%.

Proteobacteria (50.3%), Actinobacteria (19.7%), and Firmicutes (17.6%) were found to be dominants of the microbial community. The other phyla were represented to a much lesser extent: Verrucomicrobia, Chloroflexi, Planctomycea up to 4%, Myxococcota, Crenarchaeota, and Acidobacteria up to 1% of all the obtained sequences (Figure 3). At the class level, gram-negative bacteria Gammaproteobacteria and gram-positive Bacilli and Actinobacteria dominated (>10% of all obtained sequences) in the prokaryotic community (Figure 3). At the genus level, *Klebsiella, Acinetobacter* and *Verminephrobacter* represented up to 31% of all assigned nucleotide sequences in the earthworm’s gut (Figure 3).

Compared to the control, the structure of the prokaryotic community of the gut treated with pesticides changed as follows: (1) on day 7 of incubation, the relative abundance of the phylum Proteobacteria increased and the relative abundance of Actinobacteria decreased; (2) on day 14 of incubation, the relative abundance of the phyla Acidobacteria, Planctomyces, Verrucomicrobia, and Cyanobacteria increased (*p* < 0.05, Supplementary Table 3S). At the class level, on day 7 of incubation with pesticides, the relative abundance of gram-negative bacteria Gammaproteobacteria increased, while the relative abundance of Chloroflexia, Delta Proteobacteria and Anaerolineae decreased (*p* < 0.05, Supplementary Table 4S). After a 14-day incubation with pesticides, the relative abundance of the classes Ktedonobacteria, Planctomycetes, Acidobacteriia, Verrucomicrobiae and Cyanobacteriia increased (*p* < 0.05, Supplementary Table 4S). At the genus level, on day 7 of incubation with pesticides, the relative abundance of *Haliangium, Gaiella, Paenisporosarcina*, and *Oryzihumus* in *L. terrestris* gut decreased, while that of *Verminephrobacter* increased 3-fold (*p* < 0.05, Supplementary Table 5S). On day 14 of incubation of earthworms with pesticides, the relative abundance of *Candidatus Udaeobacter* and *Aquisphaera* in *L. terrestris* gut increased, while that of *Verminephrobacter* decreased 2-fold (*p* < 0.05, Supplementary Table 5S).

### Pesticide-Sensitive Bacteria of *Lumbricus terrestris* Gut

**Figure 4:**
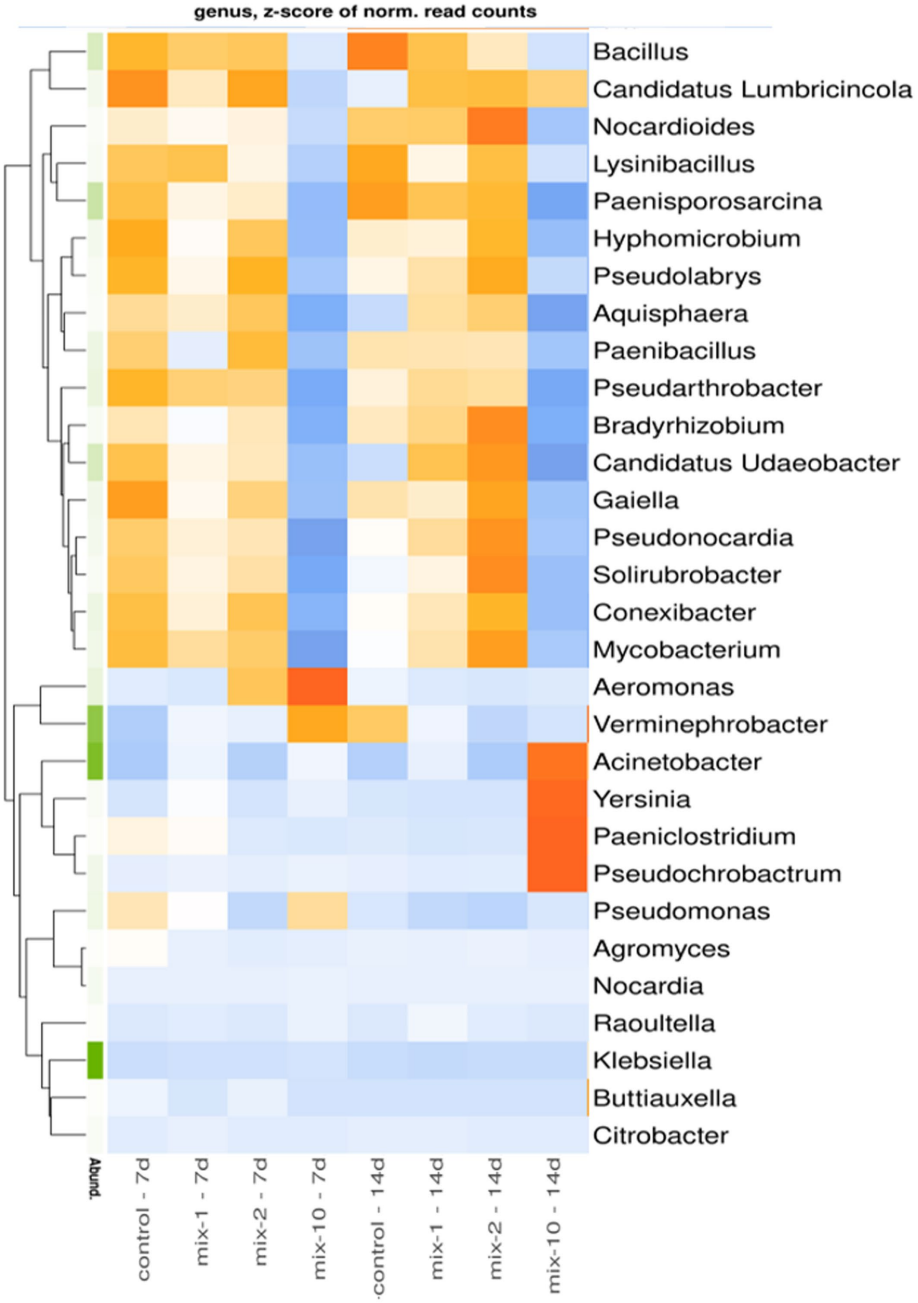
Heatmap for relative abundance of top 30 microbial taxa in prokaryotic communities of the earthworm’s guts at the genus level (*N* = 2). The data are presented as *z*-scores. Microbial genera with positive *z*-scores are marked in orange, genera with negative *z*-scores in blue. Legend: mix indicates pesticides in a mixture; 1/2/10 is the recommended, 2-fold and 10-fold pesticide application rates; 7 day/14 day is the incubation time.

The earthworm’s gut associated bacteria that considerably changed in relative abundance under pesticide exposure are shown in Figure 4. In contrast to the control variants, as well as to the variants treated with pesticides at the recommended and 2-fold application rate, in the variants treated with pesticides at a 10-fold application rate, the relative abundance of the genera *Bacillus*, *Candidatus Lumbricincola, Nocardioides, Lysinibacillus, Paenisporosarcina, Hyphomicrobium, Pseudolabrys, Aquisphaera, Paenibacillus, Pseudarthrobacter, Bradyrhizobium, Candidatus Udaeobacter, Gaiella, Pseudonocardia, Solirubrobacter, Conexibacter*, and *Mycobacterium* in *L. terrestris* gut decreased both after 7 and 14 days of the incubation experiment (Figure 4). Conversely, in the variants treated with pesticides at a 10-fold application rate, the relative abundance of *Acinetobacter, Yersinia, Paeniclostridium*, and *Pseudochrobactrum* in the earthworm’s guts increased on day 14.

### Gut Bacteria α-Diversity

**Figure 5:**
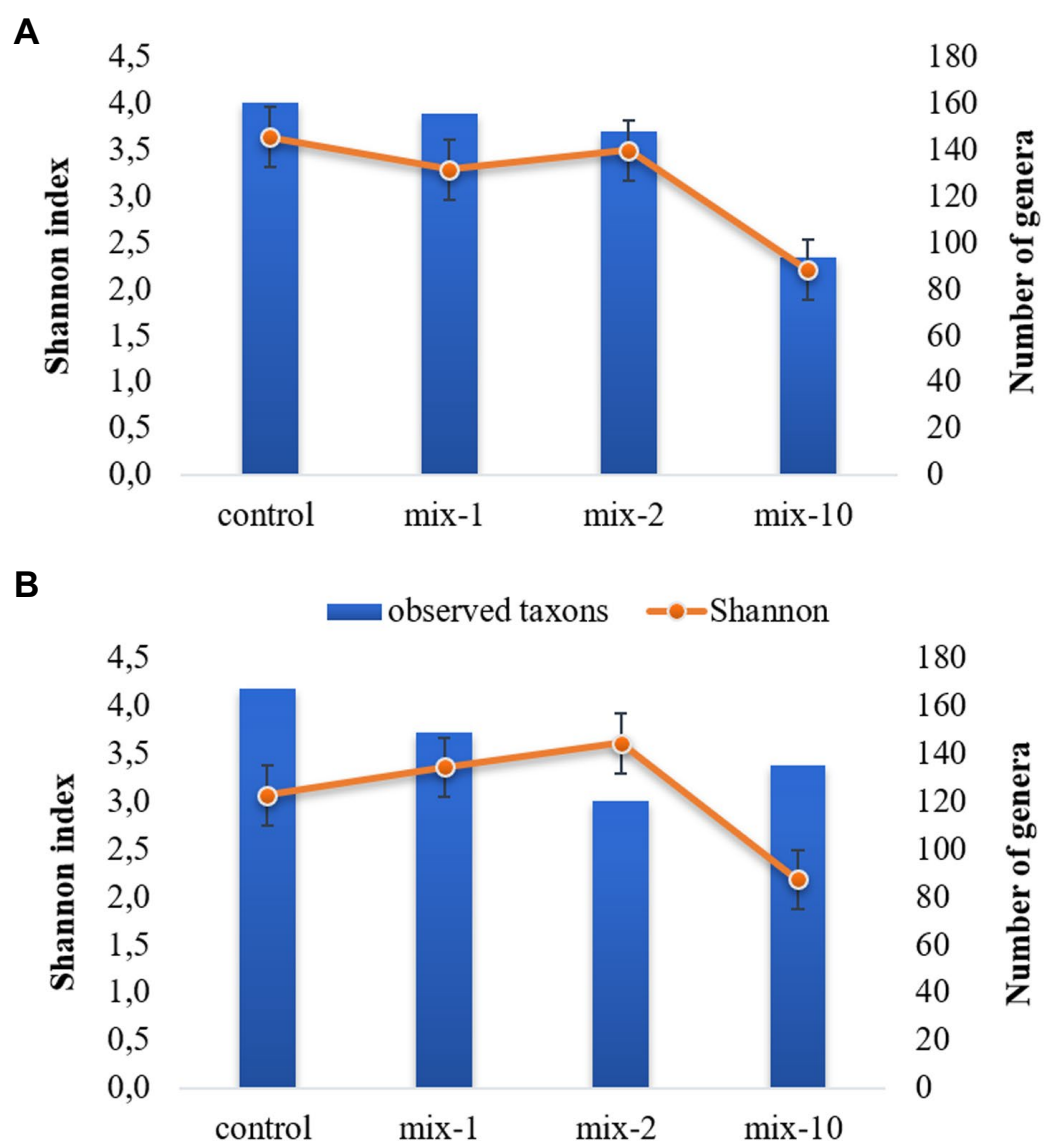
Dynamics of Shannon index and number of genera on day 7 **(A)** and day 14 **(B)** of incubation (mean ± SE). 1/2/10 is the recommended, 2-fold and 10-fold pesticide application rates.

Pesticides concentration affected the gut microbial α-diversity (Figure 5). For instance, the Shannon biodiversity index decreased from 3.6 in the control to 2.2 (*p* < 0.05) in the variants with a 10-fold application rate (Figure 5). The maximum values of the Chao1 and ACE biodiversity indexes were in the control variants, but decreased (*p* < 0.05) in the variants with a 10-fold application rate of pesticides (the data are provided in Supplementary Table 6S).

The number of identified genera decreased from 161 to 94 on day 7 after the pesticides application. However, on day 14 of the experiment, the number of identified genera increased in the variants with a 10-fold application rate compared to the variants with a 2-fold application rate. At the same time, it was lower than in the control (Figure 5).

### Gut Bacteria β-Diversity

**Figure 6:**
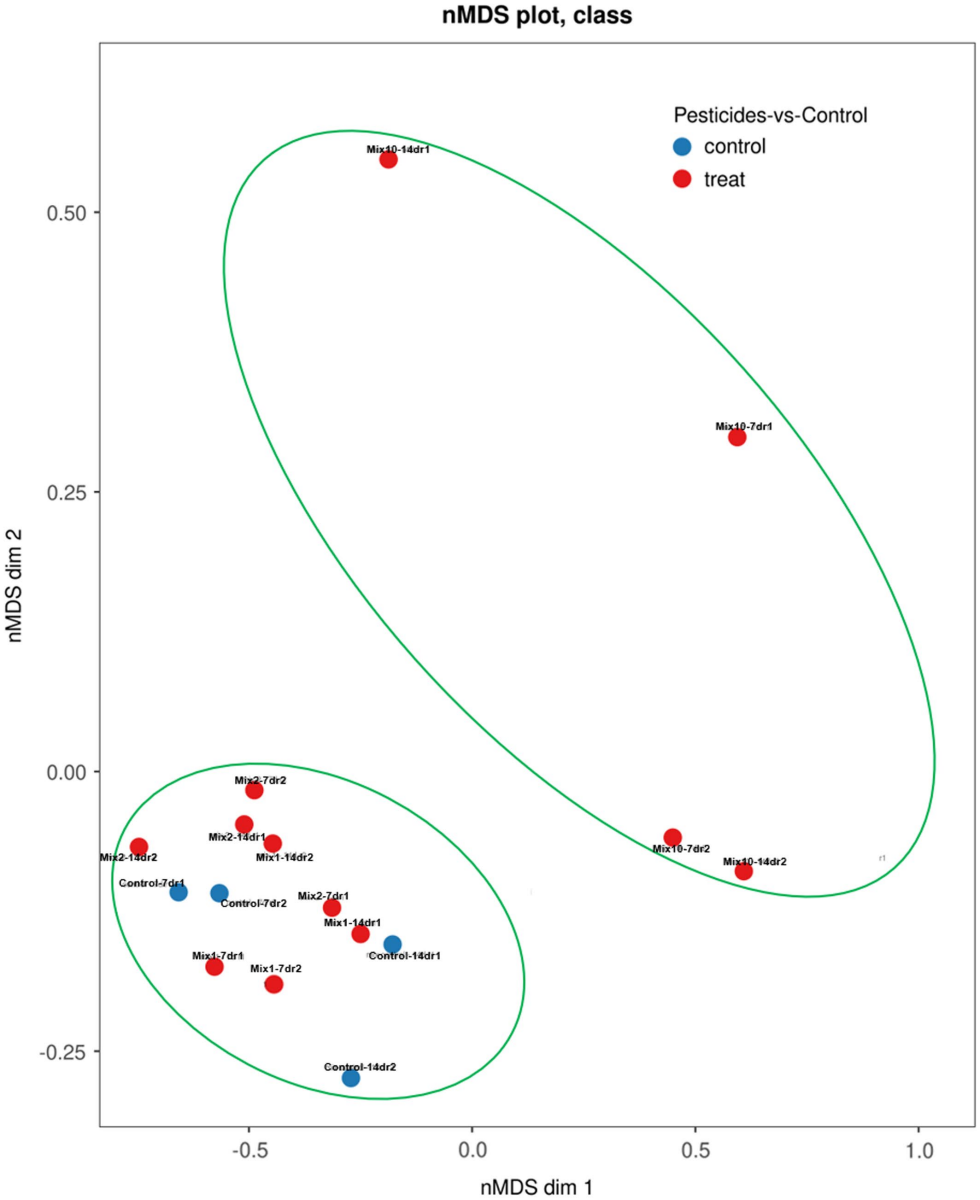
Non-metric multidimensional scaling plot of the assembly patterns of prokaryotic communities of the earthworm’s gut using the Bray–Curtis (BC) distance matrix at the genus level.

Based on the Bray-Curtis metrics and PERMANOVA tests, all samples were divided into two distinct clusters: (1) control variants, samples with the recommended and 2-fold application rates; (2) samples with a 10-fold application rate of pesticides (Figure 6).

In general, the dissimilarities in the composition of the gut bacterial communities between the samples were high. Both after 7 and 14 days of the incubation experiment, the samples with the highest concentration of pesticides (a 10-fold application rate) tended to position together in the upper right corner of the plot, in contrast to the other variants.

## Discussion

### Differences in Bacterial Communities of *Lumbricus terrestris* Gut and Soil

Our previous studies (Astaykina et al., 2020; Streletskii et al., 2022) were devoted to microbial biodiversity of Umbric Albiluvisols under pesticides treatment. We found that the pesticide exposure led to a reduction in the relative abundance of bacterial phyla Myxococcota, Bacteroidetes, Gemmatimonadetes, Proteobacteria. At the genus level pesticides increased the relative abundance of *Kitasatospora* and *Streptomyces* which could be explained by the involvement of these bacteria in the degradation of pesticides. Our current data has shown that Proteobacteria and Actinobacteria were dominant in the bacteria communities both in the earthworm’s gut and in the surrounding soil. However, in contrast to the soil, Firmicutes was also the dominant phylum in the gut. This data corresponds with previous research, where Proteobacteria, Actinobacteria, Firmucutes, Bacteroidetes, and Verrucomicrobia have been shown to be the dominant phyla in *L. terrestris* gut (Knapp et al., 2009; Pass et al., 2015; Ma et al., 2017; Meier et al., 2021). The digestive tract of an earthworm is a microaerophilic and even anaerobic zone (Byzov et al., 2015); hence, phyla Firmicutes and Actinobacteria, which are facultative and/or obligate anaerobes, dominate in the prokaryotic community of the gut (Chang et al., 2021). On the other hand, Chloroflexi, the bacteria phylum which may be among dominants in *E. fetida* gut (Liu et al., 2018), was not a dominant in *L. terrestris* gut due to the differences in burrowing and feeding habits of worms.

### Pesticides Alter the Structure of the Gut Bacteria Community

Pesticides effect on earthworms might be more complex than described by standard indicators such as LC_50,_ NOEC, body mass changes and behavioral disorders (Pelosi et al., 2014). For instance, some effects on the molecular and cellular levels (e.g., oxidative stress, DNA damage, teratogenesis) have been recently detected (Datta et al., 2016). The fungicide benomyl may cause the disruption of cell’s microtubules (Hess and Nakai, 2000) and affect the development of *E. fetida* spermatozoa (Sorour and Larink, 2001). The insecticide imidacloprid can lead to a significant reduction in *E. fetida* fecundity, as well as to a damage of the epidermal and midgut cells of the earthworms (Wang et al., 2015). The fungicide benomyl, insecticide imidacloprid and herbicide metribuzin, separately or combined, have led to changes in the diversity and structure of the soil microbiota (Astaykina et al., 2020). However, there is no data regarding the effect of these pesticides on the earthworm gut microbiota. Our results showed that these pesticides increased the relative abundance of the phyla Proteobacteria, Acidobacteria, Planctomyces, Verrucomicrobia, and Cyanobacteria in the structure of their gut prokaryotic community, while Actinobacteria decreased. A reduction in this phylum indicated the decrease in the ability to produce enzymes necessary for the cellulose, hemicellulose and other natural polymers decomposition (Vieites et al., 2010).

Previously, Chang et al. (2021) also found that the herbicide fomesafen at the recommended application rate caused significant differences in the relative abundance of Actinobacteria, Firmicutes, and Proteobacteria. Furthermore, it was found that the increased abundance of Proteobacteria can be considered as a potential marker of imbalance in the gut microbiota of many earthworm species (Shin et al., 2015; Wang et al., 2022).

At the genus level, pesticides increased the relative abundance of gram-negative *Verminephrobacter* (the phylum Proteobacteria), while the relative abundance of *Haliangium* (myxobacteria from the phylum Proteobacteria), *Gaiella* and *Oryzihumus* (gram-positive Actinobacteria)*, Paenisporosarcina* (the phylum Firmicutes) decreased after 7 days of pesticide exposure. Conversely, after 14 days of incubation the relative abundance of *Verminephrobacter* decreased, while the relative abundance of gram-negative *Candidatus Udaeobacter* (the phylum Verrucomicrobia) and *Aquisphaera* (the phylum Planctomyces) increased. The genus *Verminephrobacter* is the extracellular species-specific bacterial symbionts of Lumbricid earthworms inhabiting their nephridia, i.e., kidney-like osmoregulatory organs (Dulla et al., 2012; Lund et al., 2014). Nephridia are located next to the intestinal tract and may enter the sample during resection. Based on the results of molecular genetic analysis, bacteria of the genus *Verminephrobacter* were found to be more sensitive to the presence of pesticides in the soil than bacteria of the gut. Apparently, the pesticides application may increase the earthworms’ excretory organs activity, consequently, the excretion rate of pesticide decomposition products may increase (Wells and Laverack, 1963). *Verminephrobacter* are likely to provide enzymes or other essential co-factors for these biochemical reactions (Lund et al., 2014). However, after 14 days of the experiment, the relative abundance of *Verminephrobacter* in the prokaryotic community of lumbricid significantly decreased. This can be explained by two factors: (1) the earthworms adapted to the presence of pesticides in the soil; (2) their metabolic capacity decreased after pesticide exposure, which can be considered as an indicator of pesticide toxicity.

The verrucomicrobial genus *Candidatus Udaeobacter* was also sensitive to pesticides in the soil. Our results showed that the pesticides application increased the relative abundance of this genus. According to the previously published research, *Candidatus Udaeobacter* are the most dominant uncultured soil bacteria that can oxidize the trace gas H_2_ to generate energy and utilize nutrients due to antibiotic-driven lysis of other soil microbes (Willms et al., 2020). It is possible that pesticide treatment restructured the intestinal complex and amplified the antibiotic activity of *Candidatus Udaeobacter*. The functional role of these bacteria in the earthworm’s gut remains to be determined.

The dominant gut bacterial genera which are sensitive to pesticide treatment play an important role in organic matter decomposition and nutrient cycling both in earthworms’ guts and soils (Byzov et al., 2015). For instance, *Conexibacter* is involved in the nitrification as well as *Pseudarthrobacter* is denitrifiers (Su et al., 2019), and *Lysinibacillus* is nitrogen fixers. Therefore, a change in these bacteria abundance can affect the nitrogen cycle (Jien et al., 2021). Moreover, *Lysinibacillus sphaericus* can be used both in soil amendment in the replantation processes (Aguirre-Monroy et al., 2019) and as degraders of pesticides, in particular glyphosate (Pérez Rodríguez et al., 2019). Some *Nocardioides* species can degrade complex organic pollutants, so reducing their presence may affect the rate of natural remediation (Zhao et al., 2018).

### Effects of Pesticides Application on the Earthworm Gut Bacterial Diversity

It was found that pesticides both at the recommended application rate and at the rate increased by 10 times decreased the values of α-biodiversity indices. It is similar with the effect of other xenobiotics, such as heavy metals, microplastics, antibiotics (triclosan), pesticides (fomesafen), which significantly reduced the bacterial biodiversity in the digestive tracts of soil invertebrates (Ma et al., 2017; Chen et al., 2020; Sun et al., 2020; Chang et al., 2021). Biodiversity reduction and changing of dominants in the bacterial community of the digestive tract led to a decrease in the biochemical activity of the intestinal contents and, therefore, affected the ability of the earthworm to assimilate the substrate. The pesticides may also alter the feeding behavior, leading to changes in the composition of intestinal microbiota (Zhu et al., 2018).

The β-diversity assessment showed that bacterial complexes of *L. terrestris* gut cluster according to the application rate of pesticides. The variants with a 10-fold pesticide application rate formed a separate cluster both after 7 and 14 days of incubation. Comparison of pesticide concentrations in the variants with a 10-fold application rate with the values of acute and chronic toxicity of these pesticides for earthworms showed that even when the recommended application rate is 10-fold increased, the final concentration of the pesticide is more than 100 times lower than the values of acute and chronic toxicity of the same pesticide (0.008 mg/kg metribuzin versus LC_50_ = 427 mg/kg and NOEC >52.3 mg/kg; 0.0002 mg/kg imidacloprid versus LC_50_ = 10.7 mg/kg and NOEC = 0.178 mg/kg; 0.01 mg/kg benomyl versus LC_50_ = 5.4 mg/kg and NOEC = 1.0 mg/kg; Supplementary Table S2). Thus, the microbial community of the intestinal tract is highly sensitive to soil pollutants (Chang et al., 2021), hence, this type of research should be included in the practice of assessing the risks of pesticide application for non-target organisms.

### Pesticide-Degrading Bacteria

Numerous studies have demonstrated the crucial role of bacteria inhabiting the earthworm’s guts in the transformation of organic pollutants in the environment (Sun et al., 2020). It is known that there are different ways to transform organic pollutants in the earthworm’s gut such as the activity of transit bacteria, their free enzymes and intestinal symbionts (Byzov et al., 2015). It has been established that *Rhodococcus* and *Bacillus* bacteria from the earthworm’s intestine can degrade pesticides (Kuipa et al., 2016). In this study, we showed that pesticide treatment increases the relative abundance of Proteobacteria phylum and at the genus level, increases the relative abundance of *Acinetobacter, Pseudochrobactrum* and bacterial symbionts *Verminephrobacter*.

*Acinetobacter* and *Pseudochrobactrum* are active microbiodegraders which can degrade many xenobiotics, including pesticides (Pawar and Mali, 2014; Kafilzadeh et al., 2015; Doolotkeldieva et al., 2018; Zhan et al., 2018). The discovered fact allows us to assume that bacteria of Proteobacteria phylum which possess high hydrolytic activity can be considered as pesticide-degrading bacteria. Therefore, further studies are required to assess the physiological and biochemical potential of Proteobacteria in the digestive tract of earthworms. For instance, it would be of great interest to design primers corresponding to carboxylesterases of bioscavengers involved in pesticide detoxification (Sanchez-Hernandez et al., 2009). It is also important to determine the role of *Verminephrobacter*, as a nephridium symbiont, in the mechanisms of pesticide detoxification.

## Conclusion

In summary, based on the metagenomic analysis, pesticide-sensitive taxa, such as *Verminephrobacter, Acinetobacter, Candidatus Udaeobacter, Pseudochrobactrum* were identified in the *Lumbricus terrestris* gut. Depending on the duration of incubation, the reaction of the gut bacteria community on the presence of pesticides in the soil was different. Our results indicated that after 7 days of pesticide exposure the relative abundance of gram-negative *Verminephrobacter* increased, while the relative abundance of *Haliangium*, *Gaiella* and *Oryzihumus, Paenisporosarcina* decreased. On the contrary, after 14 days of incubation the relative abundance of *Verminephrobacter* decreased, while the relative abundance of gram-negative *Candidatus Udaeobacter* and *Aquisphaera* increased. It is possible that the gut microbiota adapted, and taxa that were initially subjected to toxic influence soon restored their abundance. We have discovered that pesticides can have a significant effect on the composition of the earthworms gut bacterial community at concentrations that were many times less than their toxicity to earthworms. Therefore, standard methods for assessing risks of pesticides application do not have enough sensitivity and the NGS methods might be recommended for a better understanding of possible changes in the soil environment under pesticides application.

## Data Availability Statement

The datasets presented in this study can be found in online repositories. The names of the repository/repositories and accession number(s) can be found at: NCBI BioProject—PRJNA797445.

## Author Contributions

AA, RS, and MM: conceptualization. RS and VG: methodology, resources, and funding acquisition. AA: software, formal analysis, data curation, writing—review and editing, visualization, and project administration. AA and GK: validation. AA and RS: investigation. MM and GK: writing—original draft preparation. MM and VG: supervision. All authors contributed to the article and approved the submitted version.

## Funding

The DNA sequencing was funded by a grant from the President of the Russian Federation (project МК-92.2021.1.5). The bioinformatic analysis was supported by the Russian Foundation for Basic Research (project 18-29-25027). The conceptualization was supported by the MSU Eurasian Center for Food Security.

## Conflict of Interest

VG was employed by the company Centre for Ecopesticides Research, LLC.

The remaining authors declare that the research was conducted in the absence of any commercial or financial relationships that could be construed as a potential conflict of interest.

## Publisher’s Note

All claims expressed in this article are solely those of the authors and do not necessarily represent those of their affiliated organizations, or those of the publisher, the editors and the reviewers. Any product that may be evaluated in this article, or claim that may be made by its manufacturer, is not guaranteed or endorsed by the publisher.

## Supplementary Material

The Supplementary Material for this article can be found online at: https://www.frontiersin.org/articles/10.3389/fmicb. 2022.853535/full#supplementary-material

## Footnotes

^1^ https://www.fao.org/faostat/en/#data/RP/visualize
