# Supplementary material for: Effects of Three Pesticides on the Earthworm Lumbricus terrestris Gut Microbiota
Source: Front Microbiol. 2022 Mar 29;13:853535. doi: 10.3389/fmicb.2022.853535 (PMC9004718; doi:10.3389/fmicb.2022.853535)
Supplement: Supplementary file 1 [file Data_Sheet_1.docx]

**SUPPLEMENTARY TABLES**

**Table 1S.** Main soil properties.

| **Parameter** | **Value** |
| --- | --- |
| Organic matter (%)  0-10 cm | 1.5±0.04 |
| рН_H2O_ | 5.6±0.2 |
| Texture class (USDA)  0-10 cm | Silt loam |
| Soil bulk density, g/cm^3^ | 1.2±0.1 |

**Table 2S.** Main properties and toxicity of benomyl, imidacloprid and metribuzin

| **Pesticide** | **Property/ Terrestrial ecotoxicology** | **Value** | **Interpretation** |
| --- | --- | --- | --- |
| Benomyl (carbendazim) | Soil degradation | DT₅₀ (typical) = 40 days | Moderately persistent |
|  | Soil adsorption | K_foc_ = 225 | Moderately mobile |
|  | Mammals | LD₅₀>10000 mg kg⁻¹ | Low |
|  |  | NOEL>10 mg kg⁻¹ | High |
|  | Birds | LD₅₀ > 2250 mg kg⁻¹ | Low |
|  |  | NOEL=26,4 mg kg⁻¹ | Moderate |
|  | Honeybees | Contact acute LD₅₀=>50 μg bee⁻¹ | Moderate |
|  |  | Oral acute LD₅₀ >100 μg bee⁻¹ | Low |
|  | Earthworms | LC₅₀ =5,4 mg kg⁻¹ | High |
|  |  | NOEC = 1,0 mg kg⁻¹ | Moderate |
| Imidacloprid | Soil degradation | DT₅₀ (typical) = 191 days | Persistent |
|  | Soil adsorption | K_foc_ = 225 | Moderately mobile |
|  | Mammals | LD₅₀=131 mg kg⁻¹ | Moderate |
|  |  | NOEL>13 mg kg⁻¹ | High |
|  | Birds | LD₅₀ =31 mg kg⁻¹ | High |
|  |  | NOEL=9,3 mg kg⁻¹ | High |
|  | Honeybees | Contact acute LD₅₀=0,081 μg bee⁻¹ | High |
|  |  | Oral acute LD₅₀ 0,0037 μg bee⁻¹ | High |
|  | Earthworms | LC₅₀ =10,7 mg kg⁻¹ | Moderate |
|  |  | NOEC >= 0,178 mg kg⁻¹ | Moderate |
| Metribuzin | Soil degradation | DT₅₀ (typical) = 7,03 days | Non-persistent |
|  | Soil adsorption | K_foc_ = 48,3 | Mobile |
|  | Mammals | LD₅₀=322 mg kg⁻¹ | Moderate |
|  |  | NOEL=2,2 mg kg⁻¹ | High |
|  | Birds | LD₅₀ =164 mg kg⁻¹ | Moderate |
|  |  | NOEL=32 mg kg⁻¹ | Moderate |
|  | Honeybees | Contact acute LD₅₀>100 μg bee⁻¹ | Low |
|  |  | Oral acute LD₅₀ >76,7 μg bee⁻¹ | Moderate |
|  | Earthworms | LC₅₀ =427 mg kg⁻¹ | Moderate |
|  |  | NOEC >52,3 mg kg⁻¹ | Moderate |

**Table 3S.** The response of bacteria phyla to pesticides application

| Taxon | **t-test p-value** | **Mann-Whitney U-test p-value** | **Spearman r** |
| --- | --- | --- | --- |
| 7 days | | | |
| Proteobacteria | 0,030 | 0,100 | 0,630 |
| Actinobacteria | 0,040 | 0,070 | -0,760 |
| 14 days | | | |
| Acidobacteriota | 0,010 | 0,400 | 0,190 |
| Planctomycetota | 0,030 | 0,400 | 0,160 |
| Verrucomicrobia | 0,050 | 0,300 | 0,200 |
| Cyanobacteria | 0,004 | 0,100 | 0,330 |

**Table 4S.** The response of bacteria classes to pesticides application

| Taxon | **t-test p-value** | **Mann-Whitney U-test p-value** | **Spearman r** |
| --- | --- | --- | --- |
| 7 days | | | |
| Gammaproteobacteria | 0,030 | 0,100 | 0,630 |
| Chloroflexia | 0,003 | 0,070 | -0,760 |
| Myxococcia | 0,040 | 0,070 | -0,760 |
| Anaerolineae | 0,002 | 0,070 | -0,760 |
| 14 days | | | |
| Ktedonobacteria | 0,020 | 0,300 | 0,230 |
| Planctomycetes | 0,030 | 0,400 | 0,160 |
| Acidobacteriae | 0,002 | 0,100 | 0,290 |
| Subgroup 5 | 0,010 | 0,300 | 0,230 |
| Verrucomicrobiae | 0,050 | 0,400 | 0,190 |
| Cyanobacteriia | 0,006 | 0,100 | 0,330 |
| Chlamydiae | 0,003 | 0,100 | 0,320 |
| Blastocatellia | 0,006 | 0,100 | 0,310 |
| Limnochordia | 0,010 | 0,400 | 0,170 |

**Table 5S.** The response of bacteria genera to pesticides application

| Taxon | **t-test p-value** | **Mann-Whitney U-test p-value** | **Spearman r** |
| --- | --- | --- | --- |
| 7 days | | | |
| *Haliangium* | 0,010 | 0,070 | -0,760 |
| *Gaiella* | 0,030 | 0,070 | -0,760 |
| *Paenisporosarcina* | 0,030 | 0,100 | -0,630 |
| *Oryzihumus* | 0,010 | 0,070 | -0,760 |
| *Verminephrobacter* | 0,050 | 0,300 | 0,500 |
| *Marmoricola* | 0,030 | 0,300 | -0,500 |
| *Microvirga* | 0,020 | 0,100 | -0,630 |
| *Verrucosispora* | 0,010 | 0,200 | -0,540 |
| *Hyphomicrobium* | 0,050 | 0,100 | -0,630 |
| *Janibacter* | 0,040 | 0,200 | -0,510 |
| *Rhodopila* | 0,009 | 0,070 | -0,760 |
| 14 days | | | |
| *Verminephrobacter* | 0,050 | 0,002 | -0,550 |
| *Aquisphaera* | 0,008 | 0,200 | 0,240 |
| *Cellulomonas* | 0,008 | 0,080 | 0,350 |
| *Parafrigoribacterium* | 0,020 | 0,100 | 0,310 |
| *1959-1* | 0,030 | 0,200 | 0,270 |
| *Candidatus Udaeobacter* | 0,040 | 0,400 | 0,190 |
| *Candidatus Xiphinematobacter* | 0,020 | 0,200 | 0,250 |
| *Catenulispora* | 0,000 | 0,030 | 0,440 |
| *Paludisphaera* | 0,000 | 0,050 | 0,400 |

**Table 6S.** Earthworm’s gut bacteria α-biodiversity indexes (genus level).

| **entry** | **reads assigned at domain** | **reads assigned at genus** | **percents** | **observed taxons** | **Shannon** | **Shannon [rarefied]** | **Chao1 (fossil) [rarefied]** | **Chao1 (vegan) [rarefied]** | **Chao std. err. (fossil) [rarefied]** | **ACE (fossil) [rarefied]** | **ACE (vegan) [rarefied]** |
| --- | --- | --- | --- | --- | --- | --- | --- | --- | --- | --- | --- |
| control - 7d r1 | 33312 | 21517 | 64,6 | 163,0 | 3,6 | 3,6 | 160,9 | 160,4 | 1,6 | 159,9 | 161,7 |
| control - 7d r2 | 28565 | 18352 | 64,2 | 158,0 | 3,7 | 3,6 | 159,9 | 159,2 | 1,9 | 158,2 | 159,6 |
| mix-1 - 7d r1 | 24736 | 16109 | 65,1 | 145,0 | 3,5 | 3,5 | 146,9 | 146,5 | 1,2 | 146,5 | 147,3 |
| mix-1 - 7d r2 | 33929 | 20024 | 59,0 | 166,0 | 3,1 | 3,1 | 169,6 | 168,8 | 2,8 | 166,2 | 170,5 |
| mix-2 - 7d r1 | 22181 | 14664 | 66,1 | 140,0 | 3,3 | 3,3 | 140,6 | 140,3 | 1,1 | 140,5 | 141,7 |
| mix-2 - 7d r2 | 30985 | 19944 | 64,4 | 156,0 | 3,7 | 3,7 | 157,4 | 157,2 | 1,0 | 157,5 | 158,6 |
| mix-10 - 7d r1 | 26904 | 13883 | 51,6 | 79,0 | 1,9 | 1,9 | 78,8 | 78,6 | 0,8 | 78,9 | 79,8 |
| mix-10 - 7d r2 | 28644 | 23524 | 82,1 | 109,0 | 2,5 | 2,5 | 112,5 | 111,6 | 2,6 | 109,5 | 112,4 |
| control - 14d r1 | 60639 | 44228 | 72,9 | 190,0 | 3,0 | 3,0 | 183,6 | 182,3 | 4,4 | 175,3 | 181,8 |
| control - 14d r2 | 39778 | 26022 | 65,4 | 144,0 | 3,2 | 3,1 | 148,6 | 147,8 | 2,6 | 145,7 | 150,3 |
| mix-1 - 14d r1 | 28033 | 19100 | 68,1 | 141,0 | 3,2 | 3,2 | 144,3 | 143,7 | 2,0 | 142,6 | 145,4 |
| mix-1 - 14d r2 | 34251 | 20803 | 60,7 | 157,0 | 3,5 | 3,5 | 161,5 | 160,1 | 3,3 | 156,1 | 158,7 |
| mix-2 - 14d r1 | 14158 | 8730 | 61,7 | 116,0 | 3,6 | 3,6 | 116,0 | 116,0 | 0,0 | 116,0 | 116,0 |
| mix-2 - 14d r2 | 18408 | 11412 | 62,0 | 125,0 | 3,6 | 3,6 | 126,1 | 125,6 | 1,0 | 125,6 | 125,9 |
| mix-10 - 14d r1 | 22188 | 18680 | 84,2 | 107,0 | 2,8 | 2,8 | 105,6 | 104,9 | 1,6 | 104,3 | 105,2 |
| mix-10 - 14d r2 | 49770 | 40137 | 80,6 | 163,0 | 1,6 | 1,6 | 180,0 | 177,0 | 9,1 | 153,9 | 184,7 |
